# A Bluetooth Indoor Positioning System Based on Deep Learning with RSSI and AoA

**DOI:** 10.3390/s25092834

**Published:** 2025-04-30

**Authors:** Yongjie Yang, Hao Yang, Fandi Meng

**Affiliations:** 1 School of Information Science and Technology, Nantong University, Nantong 226019, China; 1908110233@stmail.ntu.edu.cn (H.Y.); 2430310004@stmail.ntu.edu.cn (F.M.); 2 Nantong Research Institute for Advanced Communication Technologies, Nantong 226019, China

**Keywords:** AoA positioning, RSSI positioning, deep learning, multi-head attention, convolutional neural network, backpropagation

## Abstract

Traditional received signal strength indicator (RSSI)-based and angle of arrival (AoA)-based positioning methods are highly susceptible to multipath effects, signal attenuation, and noise interference in complex indoor environments, which significantly degrade positioning accuracy. To mitigate the impact of the above deterioration, we propose a deep learning-based Bluetooth indoor positioning system, which employs a Kalman filter (KF) to reduce the angular error in AoA measurements and utilizes a median filter (MF) and moving average filter (MAF) to mitigate fluctuations in RSSI-based distance measurements. In the deep learning network architecture, we propose a convolutional neural network (CNN)–multi-head attention (MHA) model. During the training process, the backpropagation (BP) algorithm is used to compute the gradient of the loss function and update the parameters of the entire network, gradually optimizing the model’s performance. Experimental results demonstrate that our proposed indoor positioning method achieves an average error of 0.29 m, which represents a significant improvement compared to traditional RSSI and AoA methods. Additionally, it displays superior positioning accuracy when contrasted with numerous emerging indoor positioning methodologies.

## 1. Introduction

With rapid technological advancements and continuous improvement of the public’s quality of life, the demand for indoor positioning technology is growing in various fields. Traditional outdoor positioning technologies, such as the global navigation satellite system (GNSS), can achieve the precise positioning of outdoor targets [1]. However, positioning accuracy significantly decreases within enclosed spaces due to signal obstruction and multipath phenomena; thus, indoor positioning technology has emerged to address these challenges. Received signal strength indicator (RSSI) and angle of arrival (AoA) are two of the most frequently employed interior positioning methods.

AoA estimation holds a significant position in the field of array signal processing. Classical subspace-based methods have been widely studied, including multiple-signal classification (MUSIC) [2] and the estimation of signal parameters via rotational invariance techniques (ESPRIT) [3]. In 1996, Krim summarized two decades of advancements in array signal processing, analyzing the performance and applicability of classical algorithms such as MUSIC and ESPRIT in AoA estimation. Additionally, he contrasted their assets and weaknesses in various scenarios [4]. Notably, the accuracy of these methods decreases in scenarios with low signal-to-noise ratios (SNRs) or limited snapshots. Similarly, RSSI is vital in the domains of wireless and indoor positioning. Classical RSSI-based positioning methods include the fingerprinting method, based on signal strength databases, and distance-based trilateration, which leverages path loss models for distance estimation. However, in complex indoor environments, the high variability of RSSI often leads to a decline in positioning accuracy. Researchers have proposed hybrid positioning systems that integrate these two methods to improve indoor positioning accuracy. While such fusion methods enhance accuracy somewhat, their performance remains constrained by various factors. For instance, in non-line-of-sight (NLoS) environments, AoA estimation errors increase significantly, and the variability of RSSI can result in inaccurate distance estimations [5].

Deep learning and machine learning techniques have been widely applied to IPS based on AoA or RSSI [6]. The latest deep learning models, such as artificial neural networks (ANNs), convolutional neural networks (CNNs), and graph neural networks (GNNs) [7], have demonstrated their potential in extracting features and predicting positions in the field of indoor positioning. However, these advanced models still cannot entirely eliminate the impact of environmental factors. Traditional machine learning methods, such as k-nearest neighbors (KNNs) [8], support vector machines (SVMs) [9], and random forests (RFs) [10], have also been applied to fingerprinting-based positioning.

With the rapid development of indoor positioning technologies, multi-method fusion positioning systems have gradually become a research hotspot. For example, deep learning-based WiFi fingerprinting can handle complex environments’ multipath effects and signal fluctuations [11]. The multi-sensor fusion positioning approach for indoor mobile robots using the factor graph (MSF-FG) method, which integrates inertial measurement unit (IMU) and light detection and ranging (LiDAR) data, achieves high-precision, real-time positioning in dynamic environments [12]. Additionally, indoor positioning schemes that fuse and collaborate WiFi and ultra-wideband (UWB) technologies have emerged [13]. While these fusion techniques have improved positioning accuracy, they still face numerous challenges. On the one hand, complex environments can affect measurement results, leading to a decline in positioning performance [14]. On the other hand, issues such as hardware costs, computational complexity, and the design of reasonable data fusion algorithms to fully leverage the advantages of each technology remain significant challenges [15].

This paper proposes a deep learning-based positioning system that integrates AoA and RSSI. For data processing, the system applies the Kalman filter (KF) to reduce the angular error in AoA measurements and uses a median filter (MF) and moving average filter (MAF) to address the fluctuations in RSSI-based distance measurements, thereby minimizing the impact of signal variability on the final results. A CNN–multi-head attention (MHA) model is proposed in the deep learning network architecture to extract features from angular and distance information. The model dynamically modifies the weights of input features to mitigate the impact of environmental fluctuations on positioning outcomes.

The main contributions of this work are organized as follows:

(1) We employ a KF to enhance the stability of azimuth and elevation angles. At the same time, we use MF and MAF to improve the stability of RSSI signals and the accuracy of distance estimation.

(2) We propose the CNN-MHA model. Firstly, the CNN extracts features related to orientation and distance information from the input. Then, the output of the CNN is fed into the MHA layer, where the dynamic weight adjustment capability of MHA automatically shifts the focus based on the input features. This allows the model to effectively predict the position of the signal source even when environmental changes occur, or orientation and distance information becomes distorted. Finally, the fully connected layers (FC) layer maps the output features of the MHA layer to the final positioning results. During training, the backpropagation (BP) algorithm updates the entire network’s parameters by solving the loss function’s derivative, gradually optimizing the model’s performance. Additionally, batch normalization (BN) and Dropout layers are incorporated to enhance training stability and improve generalization capabilities, while the Leaky ReLU activation mechanism is applied to address the vanishing gradient difficulty.

(3) We present a novel indoor positioning system that leverages deep learning and Bluetooth technology, integrating RSSI and AoA characteristics to enhance accuracy and robustness. Experimental results demonstrate that the system can accurately predict the position of signal sources and exhibit strong resilience in complex environments.

The rest of this paper is organized as follows. Section 2 introduces the related work. Section 3 provides a detailed explanation of data processing and the deep learning network. Section 4 presents the practical tests and simulation experiments. Finally, Section 5 concludes the paper.

## 2. Related Works

IPS usually employs filters for data preprocessing, which is a crucial step due to the complexity of indoor environments and the noise in sensor data. These techniques help suppress noise, smooth signals, and fuse data from multiple sensors. Researchers can broadly categorize them into mathematical methods and machine learning-based approaches.

Mathematical methods include the KF, particle filtering, and moving average. Particle filtering is widely adopted in warehouse environments due to its nonlinear tracking capability, whereas KF and MAF are more prevalent in office settings where linear systems dominate. The researchers can use these filters to preprocess the data, improving the accuracy of the final distance calculations [16]. For instance, in [17], the authors utilized particle filtering technology to correct vehicle position measurement errors caused by magnetic disturbances, improving measurement accuracy by leveraging collected radio signals. In [18], to enhance positioning accuracy, the researchers employed MAF as the primary algorithm while simultaneously utilizing the KF to fuse data obtained from UWB and IMU sensors. In [19], the authors employed the KF to fuse GPS, IPS, and the Inertial Navigation System (INS) data. They also utilized the Extended Kalman Filter (EKF) to linearize the nonlinear model.

Machine learning includes decision trees, long short-term memory (LSTM), neural networks (NNs), CNN, SVMs, RFs, and KNNs. Among all environments, NNs and CNN are the most commonly used. Machine learning filters can enhance data accuracy and improve predictive performance in various applications [20]. For example, in [21], to identify incidents in the refrigerated warehouse, the authors proposed an unsupervised deep-learning neural network system that utilizes distance and vibration data for detecting conditions within cold storage. In [22], the authors used the CNN to filter the input data. According to [23], given the significant impact of NLoS and multipath propagation on indoor positioning, many researchers focused their studies on mitigating or eliminating these effects. Some researchers proposed utilizing the characteristics of NN and KF to correct the errors introduced by UWB and TDOA. The authors improved the accuracy and robustness of positioning by employing deep learning based on geometric fingerprinting methods in [24], e.g., predicting initial data. Among various machine learning techniques, NNs were the most commonly used [1].

In recent years, several scholars have integrated AoA and RSSI to improve the accuracy of indoor positioning and mitigate the impact of environmental factors. For example, the authors in [6] combined AoA estimation and RSSI-based ranging with ANN. Unlike traditional ranging methods that measure the length of the direct signal path using time measurements, this method utilizes the signal propagation cycle. Successful experimental results have shown that accurate indoor distance measurement is feasible without requiring synchronization or broad signal bandwidth. In [25], authors proposed a weighted fingerprint feature-matching algorithm based on AoA and RSSI to enhance positioning accuracy. During the fingerprint database construction phase, natural discontinuity classification was utilized to identify features as fingerprint measurements. RFs were then applied to optimize the weights assigned to each attribute. The final results showed substantial enhancements in accuracy compared to KF with four base stations (KF4BSs) and KNNs. In [26], the authors first applied principal component analysis (PCA) to reduce the redundancy in RSSI measurements and used a KF to smooth AoA measurements. Subsequently, CNN was utilized for feature extraction, separately extracting deep features from RSSI and AoA measurements. The two features were then fused using a concatenation operation, followed by classification learning using a Softmax layer. Results demonstrated that this method outperformed several state-of-the-art techniques in terms of performance.

MHA is an important mechanism in deep learning and has been widely applied in natural language processing (NLP) and computer vision (CV). With the advancement of time, MHA has also been used in localization systems to enhance positioning accuracy. For example, in [27], measures were adopted to understand better and evaluate the positioning performance of the GNSS and to reduce the impact of errors on positioning. The MHA mechanism and gating operation were incorporated into the multilayer perceptron model to dynamically choose and refine features, thereby improving the model’s capacity to comprehend input data. Comparative experiments showed that the proposed method’s root mean square error (RMSE) was 39.2% lower than the latest LSTM and 17% lower than the CNN. In [28], the paper proposed an indoor localization algorithm combining MHA and practical channel state information (CSI). Through extensive experiments, the average positioning error of the algorithm was 0.71 m in the comprehensive office and 0.64 m in the laboratory.

Our proposed method integrates AoA and RSSI, utilizing KF to smooth the AoA orientation information while applying MF and MAF to process the RSSI distance information. For feature extraction, we employ CNN to extract input features and the MHA to dynamically select and weigh different input features, effectively mitigating the impact of distorted inputs on the results. During the training process, the BP algorithm is used to compute the gradient of the loss function and update the parameters of the entire network.

## 3. Methodology

**Figure 1:**
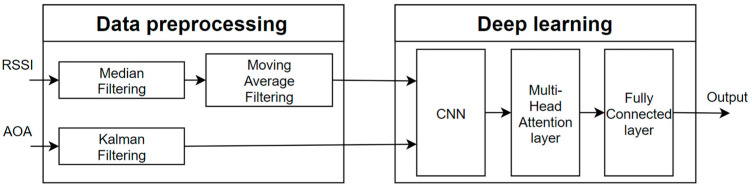
System flowchart.

In this paper, our IPS workflow, as shown in Figure 1, consists of two main components: data preprocessing and the deep learning model.

### 3.1. Data Processing

**Figure 2:**
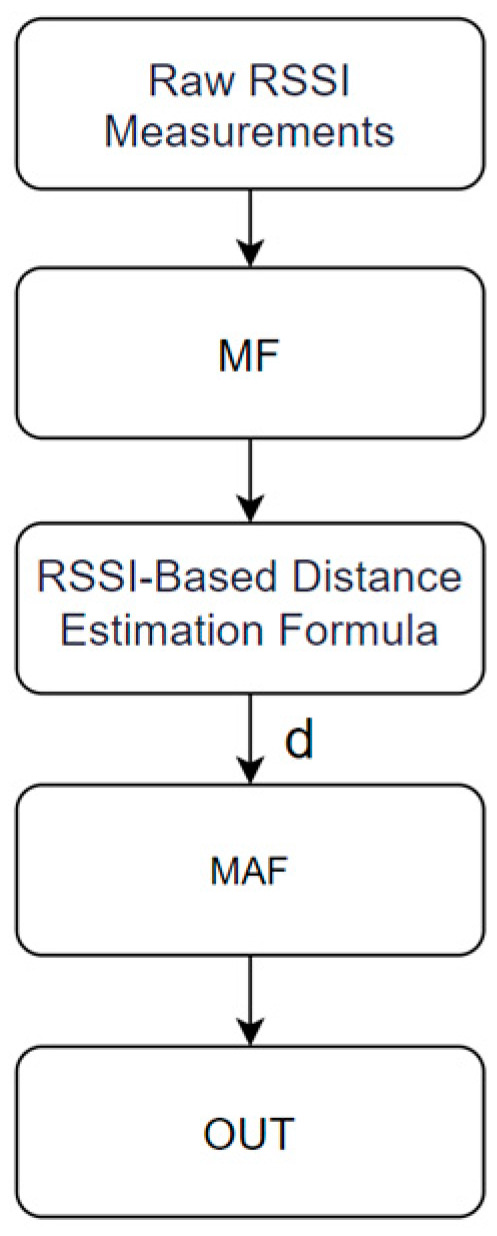
RSSI filtering process.

In the data preprocessing stage, as shown in Figure 2, We perform multiple RSSI measurements at a fixed position and first apply MF to eliminate noise, resulting in a stable median RSSI value. Subsequently, this median RSSI is converted into the corresponding distance value using an RSSI-based distance estimation formula. We repeat this process multiple times to record the distance obtained from each calculation. Finally, we subject all acquired distance values to MAF to compute a more stable and accurate final distance estimate. This method effectively enhances the accuracy and reliability of distance estimation through repeated measurements and filtering, making it applicable for indoor localization.

The KF is employed in this system to process the azimuth and elevation angle measurements provided by three AoA base stations for dynamically estimating the motion state of the target. The algorithm operates through two recursively executed phases: prediction and update. In the prediction phase, let $\theta_{azimuth,k}$ and $\theta_{elevation,k}$ denote the azimuth and elevation angles of the signal source at time k, and let $v_{azimuth,k}$ and $v_{elevation,k}$ denote the azimuth angular velocity and elevation angular velocity of the signal source at time k, respectively. The state vector is represented as

(1) $${\hat{x}}_{k}=\left[\begin{array}{c} \theta_{azimuth,k}\\ \theta_{elevation,k}\\ v_{azimuth,k}\\ v_{elevation,k} \end{array}\right].$$

The state transition matrix F and the control matrix G are represented as

(2) $$F=\left[\begin{array}{cccc} 1 & 0 & △t & 0\\ 0 & 1 & 0 & △t\\ 0 & 0 & 1 & 0\\ 0 & 0 & 0 & 1 \end{array}\right],$$

where △t is the interval between two consecutive measurements, and the off-diagonal elements of F reflect how angular velocities contribute to changes in azimuth and elevation angles over time.

(3) $$G=\left[\begin{array}{cc} \frac{1}{2}△t^{2} & 0\\ 0 & \frac{1}{2}△t^{2}\\ △t & 0\\ 0 & △t \end{array}\right].$$

By utilizing azimuth and elevation angles recorded at consecutive timestamps, the corresponding instantaneous accelerations can be computed using the second-order central difference method, represented as

(4) $$a_{\theta ,k}=\frac{\theta_{t_{k+1}}-2\theta_{t_{k}}+\theta_{t_{k-1}}}{(△t{)}^{2}},$$

where θ represents either the azimuth or elevation angle, △t is the sampling interval between consecutive angle measurements, and $t_{k-1}$, $t_{k}$ and $t_{k+1}$ denote three consecutive time instants for the angle measurements.

The azimuth and elevation accelerations calculated from the above equation, denoted as $a_{azimuth,k}$ and $a_{elevation,k}$, are subsequently employed as the system input $u_{k}$, represented as

(5) $$u_{k}=\left[\begin{array}{c} a_{azimuth,k}\\ a_{elevation,k} \end{array}\right].$$

From the information previously discussed, the state transition equation can be formulated, represented as

(6) $${\hat{x}}_{k\left|k-1\right.}=F{\hat{x}}_{k}+Gu_{k}.$$

The prediction error covariance is computed based on the uncertainty of the state estimation and is represented as

(7) $$P_{k\left|k-1\right.}=FP_{k-1\left|k-1\right.}F^{T}+Q,$$

where $P_{k\left|k-1\right.}$ is the prediction error covariance at the current time step k, $P_{k-1\left|k-1\right.}\;$ is the error covariance from the previous time step k−1, and Q is the process noise covariance matrix. In this paper, due to the possibility of the signal source being mobile, Q is dynamically adjusted, represented as follows

(8) $$Q=G\sigma_{a}^{2}G^{T},$$

where G is the control matrix and $\sigma_{a}^{2}$ is a diagonal matrix composed of the variances of the acceleration in the azimuth and elevation angles, represented as

(9) $$\sigma_{a}^{2}=\left[\begin{array}{cc} \sigma_{a_{azimuth,k}}^{2} & 0\\ 0 & \sigma_{a_{elevation,k}}^{2} \end{array}\right].$$

In the update phase, the Kalman gain is represented as

(10) $$K_{k}=P_{k\left|k-1\right.}H^{T}(HP_{k\left|k-1\right.}H^{T}+R{)}^{-1},$$

where $K_{k}$ is the Kalman gain at the current time step k, H is the observation matrix, and R is the observation noise covariance matrix. To set an appropriate R, we fix the signal source at a predetermined location within the experimental area before positioning. We measure multiple sets of azimuth and elevation angles and calculate their respective variances. The diagonal matrix R is then constructed using these two variances and can be represented as

(11) $$R=\left[\begin{array}{cc} \sigma_{\theta_{azimuth}}^{2} & 0\\ 0 & \sigma_{\theta_{elevation}}^{2} \end{array}\right].$$

The updated state equation is represented as

(12) $${\hat{x}}_{k\left|k\right.}={\hat{x}}_{k\left|k\right.-1}+K_{k}(Z_{k}-H{\hat{x}}_{k\left|k-1\right.}),$$

where ${\hat{x}}_{k\left|k\right.-1}$ is the predicted state at the current time step k, ${\hat{x}}_{k\left|k\right.}$ represents the up dated state estimate, and $Z_{k}$ is the observed value at the current time step k.

The updated error covariance is represented as

(13) $$P_{k\left|k\right.}=(I-K_{k}H)P_{k\left|k-1\right.},$$

where I is the identity matrix.

This recursive processing approach enables the system to provide more reliable real-time target state estimations, even in the presence of measurement noise and uncertainties.

### 3.2. Deep Learning Model

In the deep learning network architecture, we use CNN to extract input features, employing multiple convolutional layers to extract features from AoA and RSSI data. The extracted features are processed and then fed into the MHA layer, which dynamically selects and weights different features to reduce the impact of environmental changes. The FC layer subsequently transfers the output features of the MHA layer to the final positioning result. During the training process, the BP algorithm updates the parameters of the entire network by calculating the gradient of the loss function. BN layers are implemented to enhance training stability and accelerate model convergence by standardizing the input of each layer, thereby mitigating internal covariate shifts. Additionally, to mitigate overfitting and improve the model’s generalization performance, Dropout layers are incorporated, randomly dropping a certain proportion of neurons to reduce dependency on specific neurons. Finally, the Leaky ReLU activation function is used instead of the standard ReLU activation, alleviating the vanishing gradient problem and improving the model’s ability to represent negative features. This ultimately enables the precise output of the signal source’s position.

#### 3.2.1. Convolutional Neural Networks

**Figure 3:**
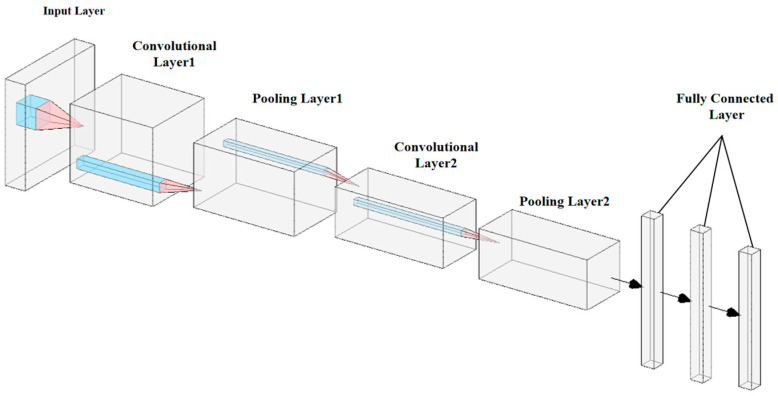
Structure of CNN.

The CNN is a deep learning architecture commonly used across numerous applications. A typical CNN comprises three main layers: convolutional, pooling, and FC. By stacking these layers together, a complete CNN architecture is established [29]. Figure 3 illustrates the configuration of a CNN.

The CNN can process pre-processed AoA and RSSI data layer by layer, effectively leveraging its powerful feature extraction capabilities. AoA data provide information regarding the azimuth and elevation angles of the signal source, while RSSI data reflect the distance relationship between the signal source and the receiver. Combining these two data types offers the model multidimensional spatial information input, enabling CNN to extract directional features from AoA data and distance-related features from RSSI data. Furthermore, downsampling operations further compress the dimensionality of the features, preserving critical spatial information while reducing computational complexity. Through integrating multiple convolutional layers and nonlinear activation functions, CNN progressively learns the complex mapping relationship from the raw data to the actual position of the signal source, thereby generating more accurate predictions for regression tasks and significantly enhancing the accuracy and robustness of indoor localization.

#### 3.2.2. Multi-Head Attention Network

The multi-head attention (MHA) mechanism is a powerful modeling method that processes input features in parallel through multiple attention heads, extracting information from different subspaces and dynamically adjusting feature weights based on the environment, improving the model’s robustness and prediction accuracy. RSSI and AoA exhibit significant sensitivity differences to environmental factors in indoor positioning tasks. For instance, RSSI is susceptible to reflection and attenuation near walls. In such cases, the MHA dynamically reduces the weight of RSSI while increasing the weight of AoA, effectively mitigating the impact of environmental changes on positioning results.

**Figure 4:**
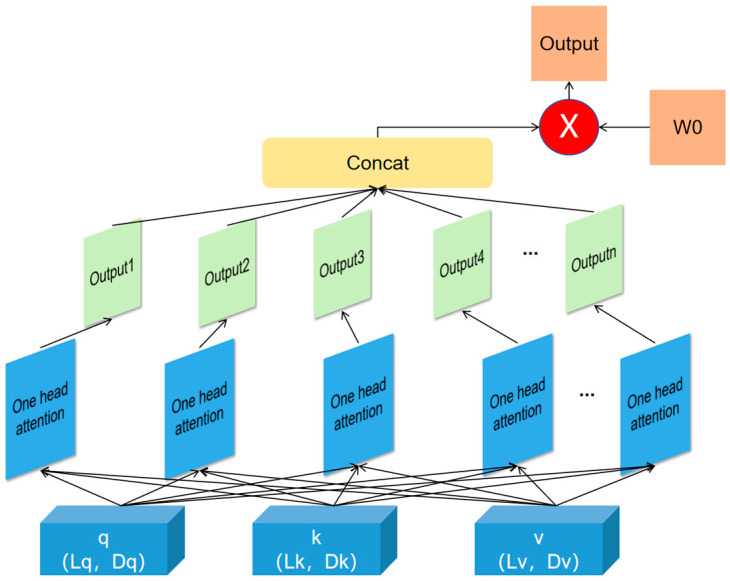
Structure of MHA.

In MHA, the final output of the CNN is processed into three components: query, key, and value, respectively. The lengths of the query, key, and value determine the dimensions of each component. Each attention head uses its own query, key, and value to compute attention scores through dot-product operations, which are then normalized using the Softmax function to obtain attention weights. Subsequently, the attention weights are used to calculate a weighted sum of the values. The outputs of each head, computed independently, represent different attention perspectives. Finally, a linear transformation concatenates and integrates the outputs of all heads, resulting in a more comprehensive feature representation. The structure of the MHA layer is shown in Figure 4.

In this paper, we utilize the MHA due to its ability to dynamically adjust the weights of angular and distance features, allowing our model to shift its attention flexibly in complex environments. This capability significantly enhances the system’s adaptability to environmental changes and improves localization accuracy. When environmental conditions change, MHA can automatically reduce reliance on distorted features while increasing attention to practical features and optimizing localization results. This dynamic adjustment mechanism enables our model to demonstrate improved robustness and accuracy across various indoor environments.

#### 3.2.3. Overview of Deep Learning Models

**Figure 5:**
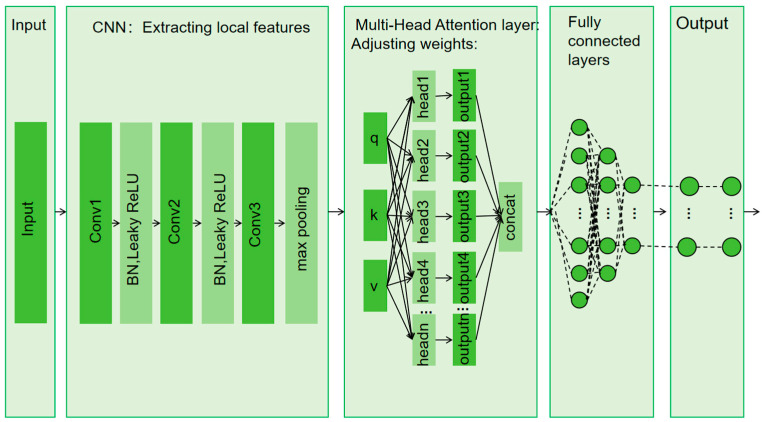
Schematic diagram of deep learning model.

The indoor positioning model based on the CNN-MHA model mainly consists of five main modules (Figure 5), in the following order: the data input module, the convolution module, the MHA module, the FC module, and the output module.

The model algorithm mainly includes the following steps:

(1) Preprocess data samples.

In the preprocessing stage, the input consists of three sets of azimuth angles, elevation angles, and distances, which total nine data points. We represent these data as a vector and then compute the outer product by multiplying the vector by its transpose to generate a 9 × 9 feature outer product matrix. This outer product transformation not only retains the original information of the input data but also significantly enhances the correlation among the features. As a result, the CNN can process the input data analogous to image processing, allowing for more effective extraction of spatial features [30].

(2) Convolution layer.

The preprocessed data passes through three convolutional layers, where key angles and distance features are extracted through the sliding operation of convolutional kernels on the input data. Each convolutional kernel consists of a weight matrix and a bias term. The weight matrix is used to extract local features, and the bias term adjusts the output values.

BN and Leaky ReLU activation functions are incorporated after each convolutional layer to mitigate overfitting.

During training, BN accelerates convergence and stabilizes the network, effectively addressing gradient vanishing and explosion. The primary operation of the BN layer is

(14) $$BN(x)=\gamma (\frac{x-\mu }{\sqrt{\sigma^{2}+\varepsilon }})+\beta ,$$

where μ is the batch mean, $\sigma^{2}\;$is the batch variance, γ and β are learnable parameters, and ε is a small constant for numerical stability.

Leaky ReLU is an activation function that enhances the training stability and efficiency of deep learning models by allowing a small, non-zero gradient for negative inputs [31]. It is defined as

(15) $$f(x)=\left\{\begin{array}{ll} x & x>0,\\ ax & x\leq 0, \end{array}\right.$$

where a is a small positive constant.

(3) MHA layer.

In order to adapt to the network structure of MHA, the data are reshaped before being fed into the MHA layer. Subsequently, the parallel attention mechanism within the MHA is employed to extract angular and distance features from the input, dynamically selecting and weighing different input information. This process enhances the precision of the final results while providing greater robustness.

(4) Full connection layer.

The final output of the MHA layer is selected as the input feature for the model to perform subsequent predictions.

In indoor localization deep learning models, FC layers are crucial in integrating features extracted by CNN and MHA mechanisms. The CNN focuses on extracting features related to angles and distance, while the MHA dynamically adjusts the weight of these features. The CNN and MHA outputs are fed into the FC layers, which perform linear transformations and nonlinear activations. By transforming high-dimensional data into lower-dimensional data, this process effectively combines information from the AoA and RSSI, capturing the complex relationships between distance and direction. This transformation enhances the accuracy and robustness of indoor localization, facilitating the practical identification and localization of signal sources in complex indoor environments.

Dropout is applied within the FC layer to reduce overfitting in deep learning models. During training, it randomly sets a fraction of neurons’ activation values to zero [32]. The mathematical expression for Dropout can be represented as

(16) $$Dropout\left(x\right)=\left\{\begin{array}{ll} x & with\;probability\;1-p,\\ 0 & with\;probability\;p, \end{array}\right.$$

where p is typically set between 0.2 and 0.5, representing the proportion of neurons to be dropped.

(5) Output layer.

In the output layer, the features processed by the FC layer are further processed to generate the predicted positional information (x, y). Typically, an error exists between the output layer’s results and the actual values, necessitating adjustments to the network parameters, including weights and biases associated with orientation and distance features. During the forward propagation phase, the input layer transmits information through nonlinear transformations across layers until the final output is obtained. Once the output is generated, the error is computed, leading to the backpropagation phase. In this phase, the loss calculation is crucial for determining gradients that reflect the discrepancy between predicted and actual positions while also considering the influence of orientation and distance features on the model output. The backpropagation process primarily involves error calculation and parameter updates [33]. If the mean squared error (MSE) is employed as the loss function, its calculation formula is as follows:

(17) $$E=\frac{1}{2}\sum_{k=1}^{m}(y_{k}-T_{k}{)}^{2}.$$

In this formula, $y_{k}$ represents the desired output and $T_{k}$ represents the actual output, with m denoting the number of neurons.

After the model training is completed, independent test data are utilized to conduct a multidimensional evaluation of the model’s performance and generalization ability, ensuring a comprehensive and objective assessment. The model parameters are summarized in Table 1.

## 4. Experimental Results and Analysis

**Figure 6:**
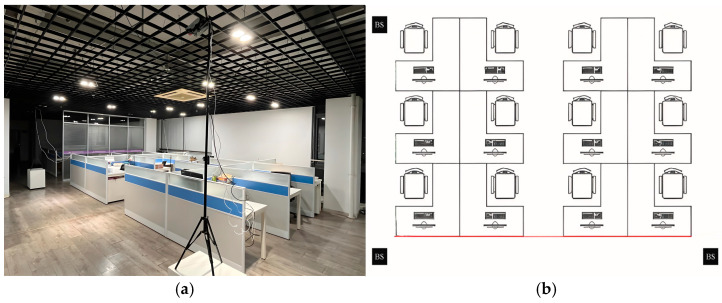
(**a**) Schematic diagram of the actual experimental site; (**b**) schematic diagram of the experimental site.

**Figure 7:**
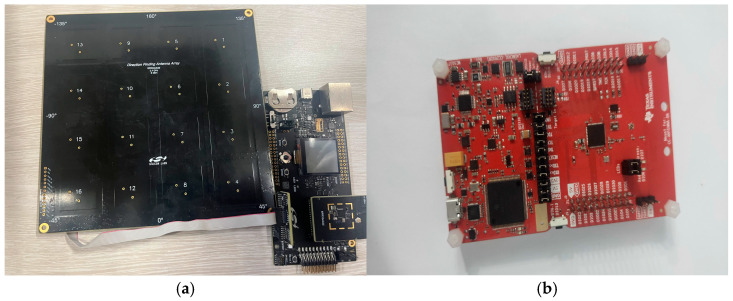
(**a**) Diagram of the RB4191A and SLWSTK6021A; (**b**) diagram of the LAUNCHXL-CC26X2R1.

The experiments in this paper were conducted in a specific indoor area with dimensions of 8 m × 5 m. As illustrated in Figure 6, the space is relatively enclosed to minimize external interference and ensure the accuracy of the localization experiments. This experiment used three sets of Bluetooth 5.1-based 4 × 4 dual-polarized antenna arrays, specifically the RB4191A antenna boards and SLWSTK6021A development boards, for AoA measurements. Three LAUNCHXL-CC26X2R1 development boards were employed as base stations to receive signals and measure RSSI for RSSI measurements, as shown in Figure 7. During the experiment, the AoA and RSSI base stations were positioned in three corners of the room, with adjacent base stations spaced 5 m and 8 m apart. This arrangement ensured accurate signal source localization and comprehensive signal coverage.

The experiment was conducted via the following steps:

1. Set up the indoor experimental environment. As shown in Figure 6, place the AoA antenna arrays and RSSI base stations at three corners and connect them to the same local area network for deployment.
2. Move a signal source within the experimental area while the PC records the real-time timestamp, azimuth angle, elevation angle, and RSSI values.
3. Process the collected data to create a dataset, dividing it into a training subset and a testing subset with a distribution of 9:1. After filtering the training data, train the deep learning model and evaluate the system’s effectiveness with the testing subset.

**Figure 8:**
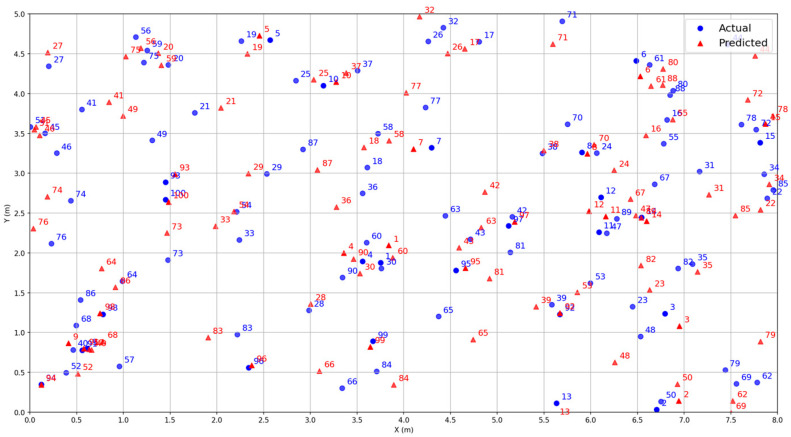
Actual measurement results.

We tested the trained model in a real-world scenario by randomly selecting 100 positions for evaluation. The test results are shown in Figure 8, where blue circles represent the actual positions of the signal source and red triangles indicate the predicted positions by the system. Figure 8 shows that the predicted coordinates are very close to the actual coordinates in most cases.

We also compared the traditional AoA-based positioning method under the same testing conditions, the AoA+Deep Learning method, and the proposed RSSI+AoA+Deep Learning method. The traditional AoA method uses the MUSIC algorithm to calculate the corresponding azimuth and elevation angles from the I/Q data received by three base stations. This angle information, combined with the known coordinates of all the base stations, is utilized in conjunction with triangulation to determine the position coordinates of the signal source. The AoA+Deep Learning method entails inputting the three sets of azimuth and elevation angle data obtained from the base stations into a deep learning model to predict the location of the signal source. The experimental results are presented in Table 2, where the first column shows the average error of the three methods, and the subsequent three columns illustrate the proportion of each positioning method within different error ranges. The proposed method achieved an average error of 0.29 m, with an accuracy of 93% for errors less than 0.4 m and 99% for errors less than 0.5 m. The results demonstrate that the proposed indoor localization method significantly improves positioning accuracy compared to the traditional and standalone AoA+Deep Learning methods.

To further evaluate the performance of the proposed method, we conducted experiments using the open-source Bluetooth 5.1 AoA+RSSI dataset released in [34]. This dataset was collected in an open indoor environment covering 110 m^2^ (13.8 m × 8 m), where four base stations were deployed in a rhombus layout around the room’s perimeter. The environment was covered by several Wi-Fi networks, mimicking a realistic indoor setting. Each base station was capable of synchronously measuring the azimuth and elevation angles of the signal source, the RSSI values in two polarization directions, and recording high-precision timestamps.

For the three-base-station model proposed in this paper, we removed the data from the east base station during preprocessing, retaining only the azimuth and elevation angles measured by the remaining three base stations. The RSSI values from the two different polarization directions at each base station were averaged, and the RSSI-based distance estimation formula was then used to calculate the relative distance between each base station and the signal source. Through this processing, we obtained a dataset compatible with our system, where each sample contains azimuth, elevation, and distance information from all three base stations for nine data points per sample.

We aim to compare the performance of different methods under various environments by generating controllable noise that conforms to the characteristics of the 2.4 GHz frequency band using MATLAB R2023a. First, we generate Gaussian white noise using MATLAB’s randn function, which exhibits independent and identically distributed properties and follows a standard normal distribution. Consequently, it ensures a uniform energy distribution in the frequency domain. The generated noise undergoes amplitude calibration to achieve the desired noise power level. Subsequently, band-limited noise is generated using a finite impulse response (FIR) bandpass filter with a cutoff frequency range of 2.4 to 2.5 GHz, and its spectral characteristics are verified through power spectral density analysis. Following this, the required noise power is calculated based on the target SNR, and the power-calibrated band-limited noise is proportionally added to the original dataset. To maintain the reproducibility of the experiments, a fixed random seed is employed during the noise generation process. This method allows precise and controllable noise injection within the 0 to 30 dB SNR range.

**Figure 9:**
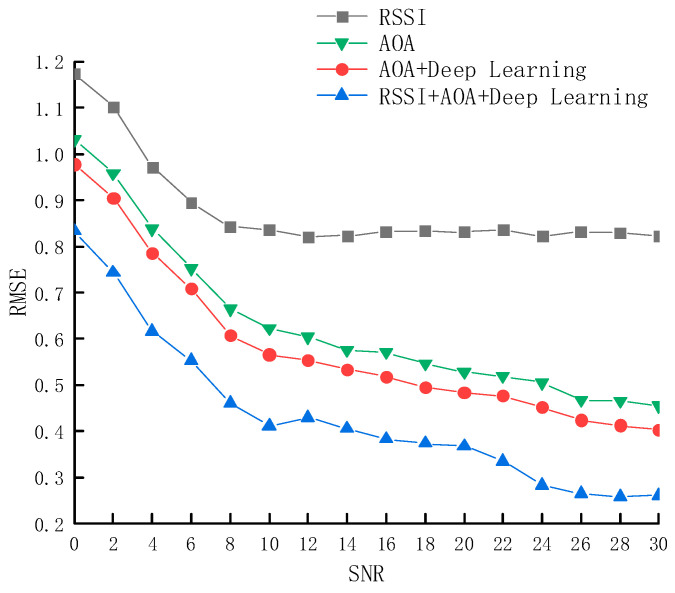
RMSE of four methods under different signal-to-noise ratios.

Figure 9 illustrates the relationship between the RMSE and the SNR, with SNR ranging from 0 to 30 dB. Under low SNR conditions (0–10 dB), the error of the RSSI-based triangulation method is significantly higher. In contrast, under high SNR conditions (20–30 dB), the RMSEs of all four methods decrease, with the deep learning method based on RSSI and AoA feature fusion consistently achieving the lowest error. Overall, the proposed method reduces the average RMSE by 0.49 m compared to the RSSI-based triangulation method, representing an improvement of 55.34%; by 0.21 m compared to AoA-based localization, representing an improvement of 34.52%; and by 0.079 m compared to the AoA+Deep Learning method, representing an improvement of 13.39%.

**Figure 10:**
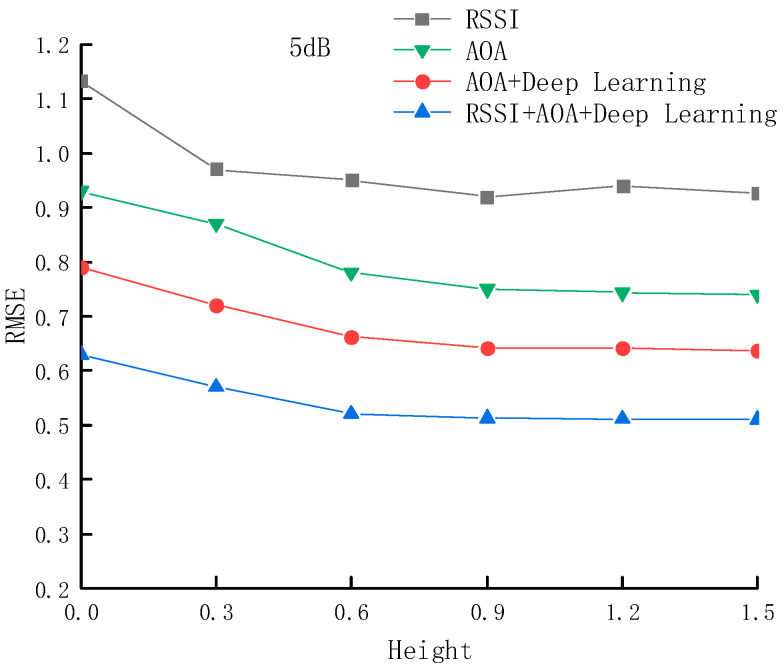
RMSE of four methods at different heights in a 5 dB SNR environment.

Figure 10 shows the variation in RMSE with height under an SNR of 5 dB for the four methods. We collected independent datasets at heights of 0 m, 0.3 m, 0.6 m, 0.9 m, 1.2 m, and 1.5 m and divided them into training and testing sets in a ratio of 9:1. We trained the deep learning model using the independent training set collected at each height and used the testing set to validate the RMSE performance of different positioning methods across the various heights. Within the height range of 0 to 0.6 m, the localization accuracy of all four methods is relatively low, with the RSSI-based triangulation method having the highest RMSE of 1.03 m. In contrast, the proposed method achieves the lowest RMSE of 0.63 m. This phenomenon is primarily attributed to signal quality degradation and enhanced multipath effects caused by ground reflections and obstacles. As the height increases beyond 0.6 m, the signal propagation path becomes more direct, and environmental noise interference decreases, leading to improved localization accuracy for all methods. Under the 5 dB SNR condition, the proposed method reduces the average RMSE by 0.33 m compared to the RSSI-based triangulation method, representing an improvement of 52.4%; by 0.19 m compared to AoA-based localization, representing an improvement of 30.6%; and by 0.06 m compared to the AoA+Deep Learning method, representing an improvement of 11.88%.

**Figure 11:**
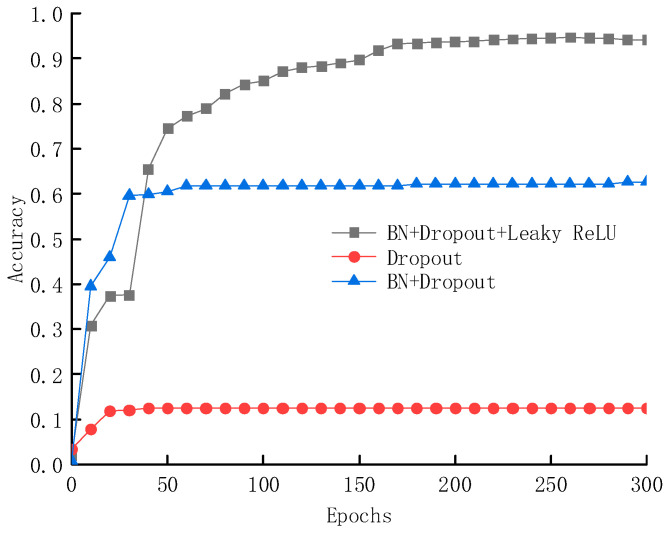
Accuracy of the testing sets under three deep learning frameworks.

**Figure 12:**
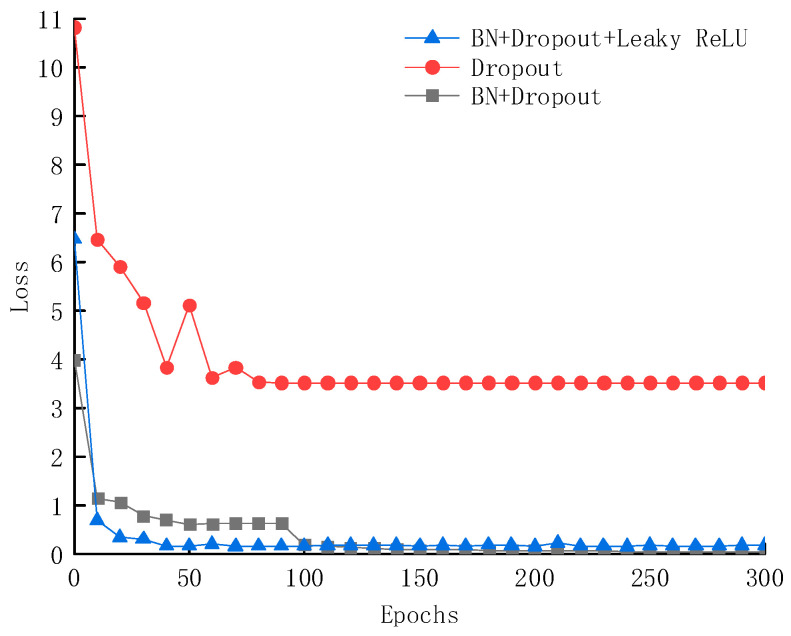
Loss rate of the testing sets under three deep learning frameworks.

The deep learning techniques adopted in this paper include BN layers, Leaky ReLU activation functions, and Dropout layers, which offer advantages such as reducing network volatility, accelerating convergence, enhancing generalization ability, and improving accuracy. The reasons for selecting these components to construct the deep learning network are illustrated in Figure 11 and Figure 12. When using only the Dropout layer, the model’s accuracy is merely 11.3%; after introducing the BN layer, the accuracy significantly increases to approximately 60%; and with the combined use of BN layers, Dropout layers, and Leaky ReLU activation functions, the network structure achieves a further improvement in accuracy, reaching 97.3%.

During the network loss training process, the proposed model rapidly reduces the loss value to 0.12 within just 50 epochs and eventually converges to 0.035. In contrast, the model using only the Dropout layer achieves a minimum loss value of only 3.8; while the model combining BN layers and Dropout layers shows some improvements, its loss value decreases slower. The experimental results demonstrate that the proposed network structure significantly outperforms other component combinations in terms of accuracy and convergence speed, validating its effectiveness and superiority.

**Figure 13:**
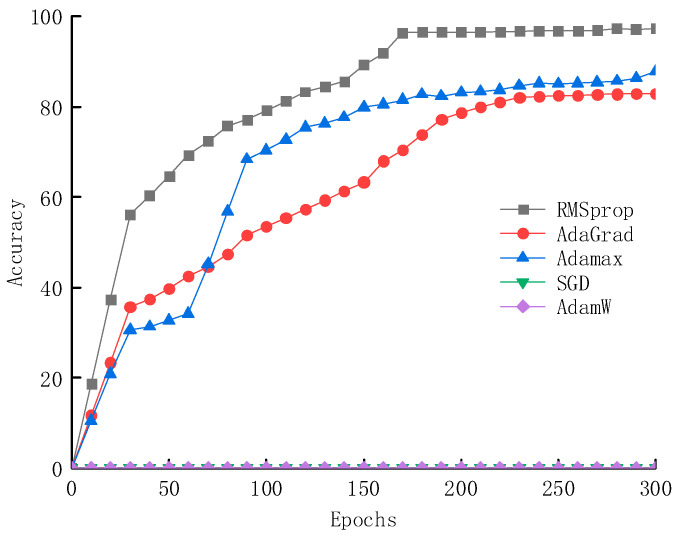
The influence of different optimizers on network accuracy.

During the construction of the deep learning network, the impact of different optimizers on the accuracy of the network is shown in Figure 13. The experimental results indicate that the model achieves the highest accuracy of 97.3% when using the RMSprop optimizer, reaching 86.7% in less than 200 epochs. In contrast, the final accuracy of the Adamax and AdaGrad optimizers is only around 85%, with slower convergence speeds. Furthermore, the SGD and AdamW optimizers perform poorly under this network framework and are unsuitable for this model.

Table 3 summarizes several recent indoor localization methods, which employ various techniques such as filters, machine learning, and sensor fusion, to achieve significant improvements in localization accuracy, robustness, and applicability. Specifically, Ref. [35] proposed a novel algorithm for integrating indoor target positioning with communication using WiFi signals for the first time. This method utilized the received signal strength (RSS) values and channel state information (CSI) values for indoor positioning, ultimately achieving a positioning error of 0.25 m. However, this method was predicated on obtaining perfect CSI values; if the CSI values were inaccurate, the positioning would also be adversely affected. In [36], the authors combined geomagnetic field strength and WiFi signals to obtain indoor localization information, offering high usability with an average error of 0.57 m. Ref. [37] proposed an indoor positioning algorithm that employed a simulated annealing (SA) algorithm and a genetic algorithm (GA) optimized neural network (SAGA-BP) to enhance the accuracy of ZigBee indoor positioning, achieving an average error of 0.75 m. In [38], the authors used CNN to process the RSS data constructed in image format, which resulted in an average error of 1.22 m. Ref. [25] introduced a weighted fingerprint feature matching algorithm based on AoA and RSSI, combined with techniques such as RFs, achieving an average error of 0.42 m. Ref. [39] proposed an indoor positioning algorithm utilizing an Adaptive Confidence-based Multi-Objective Optimization Evaluator (ACMOOE). This system enhanced positioning accuracy by adapting the impact of the two positioning techniques. The proposed ACMOOE’s positioning error was 0.45 m. Ref. [40] estimated the position of Bluetooth Low-Energy (BLE) transmitters (tags) by utilizing the characteristics of signals received from multiple anchor points (APs); additionally, the Least Squares (LS) algorithm was employed to estimate the location accurately, leading to an average error of 0.7 m. Among the various indoor positioning techniques, the method proposed in this paper shows better performance regarding average error, significantly outperforming most of the compared methods.

Notably, the last three methods mentioned in Table 3 shared similarities with the approach presented in this paper, as they were all based on the AoA and RSSI for indoor positioning. Specifically, Ref. [25] employed a NB classification method to process AoA and RSSI data for dataset generation. This paper utilized KF to process AoA data in conjunction with MF and MAF for RSSI data. Regarding feature extraction and weighting, Ref. [25] extracted feature values through NB classification and employed RFs to train feature weights. Additionally, using Multi-objective Optimization, Ref. [39] established the error propagation relationship between RSSI and AoA measurement errors and positioning errors. Similarly, Ref. [40] utilized CNN and this paper also employed CNN but introduced MHA to adjust feature weighting dynamically. In the positioning prediction phase, Ref. [25] adopted an improved KNN algorithm, while Ref. [39] employed Multi-Objective Particle Swarm Optimization (MOPSO) to obtain a Pareto optimal solution set for predicting the target position. Finally, while Ref. [40] implemented positioning through the Least Squares method, this paper transformed the high-dimensional data, dynamically adjusted by MHA, into specific positions using the FC layer. Table 4 shows the structural comparison of the three methods.

## 5. Conclusions

This paper proposes a deep learning-based Bluetooth indoor positioning system that integrates RSSI and AoA features. By combining the directional information provided by AoA with the distance information provided by RSSI, the system utilizes a deep learning model to predict the signal source’s position accurately. During the data preprocessing stage, KF reduces the error in AoA angle measurements. In contrast, MF and MAF effectively suppress fluctuations in RSSI distance measurements. The system incorporates CNN, MHA, FC, and BP in the deep learning network architecture to extract features and efficiently adjust the input feature weights. The proposed system significantly reduces the impact of environmental changes on positioning results. Experimental results demonstrate that, compared to traditional positioning methods, this system achieves significant improvements in positioning accuracy and exhibits stronger robustness in complex environments. Furthermore, compared to various advanced indoor positioning methods, this system also shows clear advantages regarding positioning accuracy. In future research, we will focus on further improving the real-time performance of the positioning system by conducting additional investigations and explorations.

## Figures and Tables

**Table 1:** Model parameters.

Module	Parameter Name	Value
Convolution Layer	Kernel Size Kernel Size	3
	Stride	1
Batch Normalization	Parameters	4, 8, 12
Pooling Layer	Pooling Parameter	2
Multi-Head Attention	Input Feature Dimension	32
	Number of Heads	4
	Queries/Keys/Values Dimensions	8
	Dropout Rate	0.1
	Feedforward Network Dimension	64
Backpropagation Network	Dropout Parameter	0.3
	Loss Function	MSELoss
	Optimizer	RMSprop
	Learning Rate	0.01

**Table 2:** The average and the proportions of errors.

Localization Method	Average Error (m)	Error ≤ 0.3 m (%)	Error ≤ 0.4 m (%)	Error ≤ 0.5 m (%)
AoA	0.94 m	0.38%	0.47%	0.51%
AoA+Deep Learning	0.64 m	0.41%	0.54%	0.62%
Proposed Method	0.29 m	0.79%	0.93%	0.99%

**Table 3:** Different position methods.

Reference Paper	Technology	Method	Filters	Data	Mean Error/m
[35]	Wi-Fi	Feature Fusion	Gaussian Filtering+KF	RSS+CSI Data	0.25
[36]	Earth’s Magnetic Field	Fingerprinting	KF	Magnetic Field Strength	0.57
[37]	ZigBee	Annealing, Gen-etic Algorithm	KF	RSSI	0.75
[38]	BLE	CNN+ Fingerprinting		RSS	1.22
[25]	AoA+RSSI	Natural Breaks+RF		AoA+RSSI	0.42
[39]	AoA+RSSI	MOPSO		AoA+RSSI	0.45
[40]	AoA+RSSI	CNN+Anchor Points		RSSI+in-phase and quadrature-phase	0.7
Proposed Method	AoA+RSSI	CNN+MHA	KF+MF+MAF	AoA+RSSI	0.29

**Table 4:** Structural comparison of four methods.

Stage	Proposed Method	[25]	[39]	[40]
Data Preprocessing	KF+MF+MAF	Natural Breaks		
Feature Extraction and Weighting	CNN+MHA	Natural Breaks+RF	Multi-objective Optimization	CNN
Positioning Prediction	FC	Improved KNN	MOPSO	Least Squares
Mean Error	0.29	0.42	0.45	0.7

## Data Availability

Data are contained within the article. The original contributions presented in this paper are included in the article, Further inquiries can be directed to the corresponding author.
